# Supplementary material for: Assessment of facility-based tuberculosis data quality in an integrated HIV/TB database in three South African districts
Source: PLOS Glob Public Health. 2022 Sep 28;2(9):e0000312. doi: 10.1371/journal.pgph.0000312 (PMC10021242; doi:10.1371/journal.pgph.0000312)
Supplement: S1 File — (PDF) [file pgph.0000312.s001.pdf]

National Tuberculosis Control Programme

TB IDENTIFICATION REGISTER

2020 Version
GW20/13

health

Department:
Health
REPUBLIC OF SOUTH AFRICA

END TB

START DATE: .... **END DATE:** ....

DISTRICT: .... **FACILITY:** .... **YEAR:** ....

NTCP TB Identification Register 1020 V9.indd 1 2020/10/06 1:51 PM

2020 Version
GW20/13

health

Department:
Health
REPUBLIC OF SOUTH AFRICA

AIM:

The main aim of this Register is to collect all the necessary information on people with TB symptoms, to assist with the following;

1. Follow-up of patients with positive results who do not come back for their results.
2. Monitoring whether all the results of specimens sent to the laboratory are returned to the facility.
3. Monitoring of the Turn-Around-Time (TAT) for results.
4. Estimating the laboratory supplies needed by the facility.
5. Follow-up of symptomatic patients referred to hospital for further investigations and final diagnosis.

COMPLETION OF THE TUBERCULOSIS IDENTIFICATION REGISTER:

1. All patients a who have one or more of the TB symptoms indicated in the TB screening tool must be recorded in this register.
2. ALL Household Contacts MUST be recorded in this register, irrespective of TB symptoms.
3. ALL HIV Positive pregnant woman enrolled in ANC for the first time MUST be recorded in this register irrespective of the TB screening outcome.
4. The 'Specimen Barcode number' is on the small barcode label on the laboratory request form. The label can be peeled off the form and affixed in the appropriate row in the register.
3. If the person is a household contact of a DS-TB or DR-TB patient, write "Y" in the upper row under the "Contact" column. In the bottom row of the same column indicate whether the index patient had drug susceptible TB (DS-TB) or drug resistant TB.
4. If not a household contact, write "No".
5. Write "Yes" at the top if the client was identified with TB symptom. Use the following codes for TB symptom to record at the bottom row. (1 = Cough for more than 2 weeks or any duration if HIV positive; 2 = Fever of more than 2 weeks; 3 = Unexplained loss of Weight/ Failure to thrive for children; 4 = Drenching night sweats; 5 = Fatigues or less playful for children.) If a client present with more than one symptom use comma (;) to separate. Write No if no TB symptom was identified but client meet criteria to be investigated.
6. Write "Yes" If patient is a known diabetic on treatment, "No" if a patient has tested before and told they are not diabetic, "unknown" if patient does not know or never tested before for diabetes.
7. Write "Pos" if patient is a known HIV positive person, "Neg" if patient tested negative in the past year, "Unk" if HIV status is unknown or patient has never tested before.
8. Write "Yes" if patient has been confirmed as pregnant, "No" if patient says she is not pregnant.
9. Write "Pos" if a patient has had a positive COVID -19 test in the past 2 weeks , "No" if the patient has tested negative for COVD-19 in the past 2 weeks or "unk" if the patient has not tested in the past 2 weeks.
10. Use the Column "Xpert" to record the results of the Xpert test. Use the top row to record the First test taken. Where a second Xpert specimen is collected following an unsuccessful first Xpert test (leaked, indeterminate, contaminated specimen) this must be recorded in the second row in the Xpert test.
11. Where a pretreatment sputum specimen for baseline smear microscopy is collected following an Xpert positive result, this must be entered in the column marked as "Smear Microscopy". Record the results of the test in top row (Pos, Neg, Scanty, not done/ no result), and record grading of smear positive results in the bottom row (i.e. +/++/+++).
Note: *All positive results must be recorded with a RED pen, and all other results recorded with a black pen in the register.*
12. The date the specimen was collected must be entered in the "Date specimen collected" column and the date the results were received at the facility entered in the "Date Results received" column.
Note: *The TAT is calculated from the time the sputum was collected to the time the results were received in the facility NOT the date on the laboratory result report.*
13. Indicate Rifampicin sensitivity based on the Xpert lab results. Record "R" if Rifampicin resistance and "S" if Rifampicin Sensitive.
14. For non Bacteriological Investigations, record the date in which investigation was conducted at the top and results at the bottom. If "Other tests" write the test used to make a diagnosis under "Remarks" column.
15. The treatment start date must be entered in the column "TB confirmed clients" in the correct format. The TB Registration number must be recorded in the "Remarks Column".
16. If the patient died before treatment was started, tick in the column "Died before treatment start" and if the date of death is known it must be entered.
17. If the patient is lost to follow-up, tick in the column "Lost to follow up" and explain under "Remarks" column the outcomes of the tracing. The definition of loss to follow up in this case is a patient who missed an appointment for the results, traced but not found in two weeks.
18. When other tests such as Culture, LPA and DST are conducted, the specimen collection date should be recorded under column "Date specimen collected". The results should be entered under the column "Results" and "Resistance" columns. If tests other than bacteriological tests are conducted, these must be recorded under the column "Non-Bacteriological Test". The Date of the test should be recorded in the bottom row, the test result should be recorded in the top row.
19. If patient is diagnosed with DR-TB, the treatment start date must be entered in the "Patient diagnosed with TB/ DR-TB" column. The MDR-TB treatment site where the patient was referred must be documented under the "Remarks" column.
20. If patient with DR-TB "died" or "lost to follow up" before treatment is started capture in the register as outlined in 16 and 17 above.
21. For all contacts (irrespective of HIV status) and PLHIV found not to have TB (negative test results) and started on TPT, the TPT start date must be entered in the column "TPT start date".
22. At the end of each page the totals must calculated and entered in the last row labelled "totals".
23. The person completing the register must write his/her name and sign at the bottom of each page.
26. The person who checks the data for correctness and completeness must write his/her name and sign at the bottom of each page.

COMPLETION OF THE DATA SUMMARY SHEET:

1. At the end of each register is a copy of the data summary sheet that needs to be completed at the end of each month.
2. The data elements from the summary sheet must be entered in the Monthly Data Input Form.
3. At the end of each quarter the data must be collated and submitted to the District as part of the quarterly reports.
4. The data summary sheets remain in the facility for audit/ data verification purposes.

health
Department:
Health
REPUBLIC OF SOUTH AFRICA

TUBERCULOSIS IDENTIFICATION REGISTER

District: ....

							TB symptoms Identified	Risk Groups					TB Investigations	
													Bacteriological investigation	
													BASELINE TESTS	
Specimen Barcode Number[1]	Patient Folder Number	Surname (Top row) / First name(s) (Bottom row)	Date of birth (Top row) DD / MM / YYYY / Age (Bottom row)	Gender M / F	Physical Home Address[2] / Land Mark	Telephone / Cellphone	Yes / No / If Yes (Code)[3]	Contact (Y / N) (Top row) / DS/ DR- TB / unk (Bottom row)	HIV Status (Pos / Neg / Unk)	Pregnant (Y / N)	Diabetes Mellitus (Y / N / Unk)	COVID-19 (Pos / Neg / Unk)	Date Specimen collected[4] DD / MM / YYYY	Date test Result received[5] DD / MM / YYYY
													1	1
													2	2
													1	1
													2	2
													1	1
													2	2
													1	1
													2	2
													1	1
													2	2
													1	1
													2	2
													1	1
													2	2
													1	1
													2	2
													1	1
													2	2
													1	1
													2	2
TOTALS:									Total number positive:	Total number pregnant:	Total number Diabetic:	Total number positive:		

1. Place Barcode sticker from the laboratory form / write Barcode number eg. AAGX8459

2. Full detailed address of where the patient lives or can be reached during the day.

3. Use the coding on the instruction cover for TB symptoms (i.e. 1= Cough)

4. Write down a date the patient produced specimen

5: Write date when the test results were received by facility

Sub District: .... Facility Name: .... 2020 Version GW20/13

TB Investigations												TB Confirmed Clients (DS-TB and DR-TB)			TPT Start Date[8] (for contacts, PLHIV) DD / MM / YYYY	REMARKS
Bacteriological investigation								Non- Bacteriological Investigations								
**Xpert** (1st Test)	**Smear Microscopy** (Pos, Neg, Scanty, not done / no result)	**TEST RESULT TURN AROUND TIME**[6] (Tick)		**Rif Susceptibility**	**TB CULTURE / LPA / DST**			**X-Ray** DD / MM / YYYY	**U-LAM** DD / MM / YYYY	**Mantoux** DD / MM / YYYY	**Other Tests** DD / MM / YYYY	**Date Treatment Started**[7] DD / MM / YYYY	**Died before Treatment Start** (Tick)	**Loss to follow up** (Tick)		
**Xpert** (2nd test if any)	**Grading** (bottom row)	< 48hrs	> 48hrs	R / S	**Date specimen collected** DD / MM / YYYY	**Result** (Pos / Neg)	**Resistance** (R / H)	Result	Result	Result	Result					
1		1	1													
2		2	2													
1		1	1													
2		2	2													
1		1	1													
2		2	2													
1		1	1													
2		2	2													
1		1	1													
2		2	2													
1		1	1													
2		2	2													
1		1	1													
2		2	2													
1		1	1													
2		2	2													
1		1	1													
2		2	2													
Total Xpert Positive:				Total Rif Resistance		Total tested Positive:	Total MDR-TB:	Total number suggestive:	Total number testedpositive:	Total number reactive:	Total:	Total started Rx:	Total died:	Total not on Rx:	Total started TPT:	

6. Tick whether sputum results were back within 48hrs.

7. Write date on which the patient was initiated on Treatment.

8. Write a date for TPT start.

Complied by: .... Signature: .... Date: .... Verified by: .... Signature: .... Date: ....

health
Department:
Health
REPUBLIC OF SOUTH AFRICA

TUBERCULOSIS IDENTIFICATION REGISTER

District: ....

								Risk Groups					TB Investigations	
													Bacteriological investigation	
													BASELINE TESTS	
**Specimen Barcode Number[1]**	**Patient Folder Number**	**Surname** (Top row) **First name(s)** (Bottom row)	**Date of birth** (Top row) DD / MM / YYYY **Age** (Bottom row)	**Gender M / F**	**Physical Home Address[2] / Land Mark**	**Telephone / Cellphone**	**TB symptoms Identified** **Yes / No** **If Yes (Code)[3]**	**Contact (Y / N)** (Top row) **DS/ DR- TB / unk** (Bottom row)	**HIV Status** (Pos / Neg / Unk)	**Pregnant** (Y / N)	**Diabetes Mellitus** (Y / N / Unk)	**COVID-19** (Pos / Neg / Unk)	**Date Specimen collected[4]** DD / MM / YYYY	**Date test Result received[5]** DD / MM / YYYY
													1	1
													2	2
													1	1
													2	2
													1	1
													2	2
													1	1
													2	2
													1	1
													2	2
													1	1
													2	2
													1	1
													2	2
													1	1
													2	2
													1	1
													2	2
													1	1
													2	2
TOTALS:									Total number positive:	Total number pregnant:	Total number Diabetic:	Total number positive:		

1. Place Barcode sticker from the laboratory form / write Barcode number eg. AAGX8459

2. Full detailed address of where the patient lives or can be reached during the day.

3. Use the coding on the instruction cover for TB symptoms (i.e. 1= Cough)

4. Write down a date the patient produced specimen

5: Write date when the test results were received by facility

Sub District: .... Facility Name: .... 2020 Version GW20/13

TB Investigations													TB Confirmed Clients (DS-TB and DR-TB)			TPT Start Date[8] (for contacts, PLHIV) DD / MM / YYYY	REMARKS
Bacteriological investigation									Non- Bacteriological Investigations								
**Xpert** (1st Test)	**Smear Microscopy** (Pos, Neg, Scanty, not done / no result)	**TEST RESULT TURN AROUND TIME[6]** (Tick)		**Rif Susceptibility**	**TB CULTURE / LPA / DST**				**X-Ray** DD / MM / YYYY	**U-LAM** DD / MM / YYYY	**Mantoux** DD / MM / YYYY	**Other Tests** DD / MM / YYYY	**Date Treatment Started[7]** DD / MM / YYYY	**Died before Treatment Start** (Tick)	**Loss to follow up** (Tick)		
**Xpert** (2nd test if any)	**Grading** (bottom row)	< 48hrs	> 48hrs	R / S	**Date specimen collected** DD / MM / YYYY	**Result** (Pos / Neg)	**Resistance** (R / H)		Result	Result	Result	Result					
1		1	1														
2		2	2														
1		1	1														
2		2	2														
1		1	1														
2		2	2														
1		1	1														
2		2	2														
1		1	1														
2		2	2														
1		1	1														
2		2	2														
1		1	1														
2		2	2														
1		1	1														
2		2	2														
1		1	1														
2		2	2														
1		1	1														
2		2	2														
Total Xpert Positive:				Total Rif Resistance		Total tested Positive:	Total MDR-TB:		Total number suggestive:	Total number testedpositive:	Total number reactive:	Total:	Total started Rx:	Total died:	Total not on Rx:	Total started TPT:	

6. Tick whether sputum results were back within 48hrs.

7. Write date on which the patient was initiated on Treatment.

8. Write a date for TPT start.

Complied by: .... Signature: .... Date: ....

Verified by: .... Signature: .... Date: ....

health
Department:
Health
REPUBLIC OF SOUTH AFRICA

TUBERCULOSIS IDENTIFICATION REGISTER

District: ....

Specimen Barcode Number[1]	Patient Folder Number	Surname (Top row) First name(s) (Bottom row)	Date of birth (Top row) DD / MM / YYYY	Gender M / F	Physical Home Address[2] / Land Mark	Telephone / Cellphone	TB symptoms Identified	Risk Groups					TB Investigations	
							Yes / No	Contact (Y / N) (Top row)	HIV Status (Pos / Neg / Unk)	Pregnant (Y / N)	Diabetes Mellitus (Y / N / Unk)	COVID-19 (Pos / Neg / Unk)	Bacteriological investigation	
			Age (Bottom row)				If Yes (Code)[3]	DS/ DR- TB / unk (Bottom row)					BASELINE TESTS	
													Date Specimen collected[4] DD / MM / YYYY	Date test Result received[5] DD / MM / YYYY
													1	1
													2	2
													1	1
													2	2
													1	1
													2	2
													1	1
													2	2
													1	1
													2	2
													1	1
													2	2
													1	1
													2	2
													1	1
													2	2
													1	1
													2	2
													1	1
													2	2
TOTALS:									Total number positive:	Total number pregnant:	Total number Diabetic:	Total number positive:		

1. Place Barcode sticker from the laboratory form / write Barcode number eg. AAGX8459

2. Full detailed address of where the patient lives or can be reached during the day.

3. Use the coding on the instruction cover for TB symptoms (i.e. 1= Cough)

4. Write down a date the patient produced specimen

5: Write date when the test results were received by facility

Sub District: .... Facility Name: .... 2020 Version GW20/13

TB Investigations													TB Confirmed Clients (DS-TB and DR-TB)			TPT Start Date[8] (for contacts, PLHIV) DD / MM / YYYY	REMARKS
Bacteriological investigation								Non- Bacteriological Investigations									
**Xpert** (1st Test)	**Smear Microscopy** (Pos, Neg, Scanty, not done / no result)	**TEST RESULT TURN AROUND TIME[6]** (Tick)		**Rif Susceptibility**	**TB CULTURE / LPA / DST**			**X-Ray** DD / MM / YYYY	**U-LAM** DD / MM / YYYY	**Mantoux** DD / MM / YYYY	**Other Tests** DD / MM / YYYY	**Date Treatment Started[7]** DD / MM / YYYY	**Died before Treatment Start** (Tick)	**Loss to follow up** (Tick)			
**Xpert** (2nd test if any)	**Grading** (bottom row)	< 48hrs	> 48hrs	R / S	**Date specimen collected** DD / MM / YYYY	**Result** (Pos / Neg)	**Resistance** (R / H)	Result	Result	Result	Result						
1		1	1														
2		2	2														
1		1	1														
2		2	2														
1		1	1														
2		2	2														
1		1	1														
2		2	2														
1		1	1														
2		2	2														
1		1	1														
2		2	2														
1		1	1														
2		2	2														
1		1	1														
2		2	2														
1		1	1														
2		2	2														
1		1	1														
2		2	2														
Total Xpert Positive:				Total Rif Resistance		Total tested Positive:	Total MDR-TB:	Total number suggestive:	Total number testedpositive:	Total number reactive:	Total:	Total started Rx:	Total died:	Total not on Rx:	Total started TPT:		

6. Tick whether sputum results were back within 48hrs.

7. Write date on which the patient was initiated on Treatment.

8. Write a date for TPT start.

Complied by: .... Signature: .... Date: .... Verified by: .... Signature: .... Date: ....

health
Department:
Health
REPUBLIC OF SOUTH AFRICA

TUBERCULOSIS IDENTIFICATION REGISTER

District: ....

Specimen Barcode Number[1]	Patient Folder Number	Surname (Top row) First name(s) (Bottom row)	Date of birth (Top row) DD / MM / YYYY	Gender M / F	Physical Home Address[2] / Land Mark	Telephone / Cellphone	TB symptoms Identified	Risk Groups					TB Investigations	
							Yes / No	Contact (Y / N) (Top row)	HIV Status (Pos / Neg / Unk)	Pregnant (Y / N)	Diabetes Mellitus (Y / N / Unk)	COVID-19 (Pos / Neg / Unk)	Bacteriological investigation	
													BASELINE TESTS	
			Age (Bottom row)				If Yes (Code)[3]	DS/ DR- TB / unk (Bottom row)					Date Specimen collected[4] DD / MM / YYYY	Date test Result received[5] DD / MM / YYYY
													1	1
													2	2
													1	1
													2	2
													1	1
													2	2
													1	1
													2	2
													1	1
													2	2
													1	1
													2	2
													1	1
													2	2
													1	1
													2	2
													1	1
													2	2
													1	1
													2	2
TOTALS:									Total number positive:	Total number pregnant:	Total number Diabetic:	Total number positive:		

1. Place Barcode sticker from the laboratory form / write Barcode number eg. AAGX8459

2. Full detailed address of where the patient lives or can be reached during the day.

3. Use the coding on the instruction cover for TB symptoms (i.e. 1= Cough)

4. Write down a date the patient produced specimen

5: Write date when the test results were received by facility

NTCP TB Identification Register 1020 V9.indd 10 2020/10/06 1:51 PM

Sub District: .... Facility Name: ....

TB Investigations												TB Confirmed Clients (DS-TB and DR-TB)			TPT Start Date[8] (for contacts, PLHIV) DD / MM / YYYY	REMARKS
Bacteriological investigation								Non- Bacteriological Investigations								
**Xpert** (1st Test)	**Smear Microscopy** (Pos, Neg, Scanty, not done / no result)	**TEST RESULT TURN AROUND TIME**[6] (Tick)		**Rif Susceptibility**	**TB CULTURE / LPA / DST**			**X-Ray** DD / MM / YYYY	**U-LAM** DD / MM / YYYY	**Mantoux** DD / MM / YYYY	**Other Tests** DD / MM / YYYY	**Date Treatment Started**[7] DD / MM / YYYY	**Died before Treatment Start** (Tick)	**Loss to follow up** (Tick)		
**Xpert** (2nd test if any)	**Grading** (bottom row)	< 48hrs	> 48hrs	R / S	**Date specimen collected** DD / MM / YYYY	**Result** (Pos / Neg)	**Resistance** (R / H)	Result	Result	Result	Result					
1		1	1													
2		2	2													
1		1	1													
2		2	2													
1		1	1													
2		2	2													
1		1	1													
2		2	2													
1		1	1													
2		2	2													
1		1	1													
2		2	2													
1		1	1													
2		2	2													
1		1	1													
2		2	2													
1		1	1													
2		2	2													
1		1	1													
2		2	2													
Total Xpert Positive:				Total Rif Resistance		Total tested Positive:	Total MDR-TB:	Total number suggestive:	Total number testedpositive:	Total number reactive:	Total:	Total started Rx:	Total died:	Total not on Rx:	Total started TPT:	

6. Tick whether sputum results were back within 48hrs.

7. Write date on which the patient was initiated on Treatment.

8. Write a date for TPT start.

Complied by: .... Signature: .... Date: .... Verified by: .... Signature: .... Date: ....

health
Department:
Health
REPUBLIC OF SOUTH AFRICA

TUBERCULOSIS IDENTIFICATION REGISTER

District: ....

Specimen Barcode Number[1]	Patient Folder Number	Surname (Top row) / First name(s) (Bottom row)	Date of birth (Top row) DD / MM / YYYY	Gender M / F	Physical Home Address[2] / Land Mark	Telephone / Cellphone	TB symptoms Identified	Risk Groups					TB Investigations	
							Yes / No	Contact (Y / N) (Top row)	HIV Status (Pos / Neg / Unk)	Pregnant (Y / N)	Diabetes Mellitus (Y / N / Unk)	COVID-19 (Pos / Neg / Unk)	Bacteriological investigation	
			Age (Bottom row)				If Yes (Code)[3]	DS/ DR- TB / unk (Bottom row)					BASELINE TESTS	
													Date Specimen collected[4] DD / MM / YYYY	Date test Result received[5] DD / MM / YYYY
													1	1
													2	2
													1	1
													2	2
													1	1
													2	2
													1	1
													2	2
													1	1
													2	2
													1	1
													2	2
													1	1
													2	2
													1	1
													2	2
													1	1
													2	2
													1	1
													2	2
TOTALS:									Total number positive:	Total number pregnant:	Total number Diabetic:	Total number positive:		

1. Place Barcode sticker from the laboratory form / write Barcode number eg. AAGX8459

2. Full detailed address of where the patient lives or can be reached during the day.

3. Use the coding on the instruction cover for TB symptoms (i.e. 1= Cough)

4. Write down a date the patient produced specimen

5: Write date when the test results were received by facility

Sub District: .... Facility Name: ....

2020 Version
GW20/13

TB Investigations												TB Confirmed Clients (DS-TB and DR-TB)			TPT Start Date[8] (for contacts, PLHIV) DD / MM / YYYY	REMARKS
Bacteriological investigation								Non- Bacteriological Investigations								
**Xpert** (1st Test)	**Smear Microscopy** (Pos, Neg, Scanty, not done / no result)	**TEST RESULT TURN AROUND TIME[6]** (Tick)		**Rif Susceptibility**	**TB CULTURE / LPA / DST**			**X-Ray** DD / MM / YYYY	**U-LAM** DD / MM / YYYY	**Mantoux** DD / MM / YYYY	**Other Tests** DD / MM / YYYY	**Date Treatment Started[7]** DD / MM / YYYY	**Died before Treatment Start** (Tick)	**Loss to follow up** (Tick)		**REMARKS**
**Xpert** (2nd test if any)	**Grading** (bottom row)	< 48hrs	> 48hrs	R / S	**Date specimen collected** DD / MM / YYYY	**Result** (Pos / Neg)	**Resistance** (R / H)	Result	Result	Result	Result					
1		1	1													
2		2	2													
1		1	1													
2		2	2													
1		1	1													
2		2	2													
1		1	1													
2		2	2													
1		1	1													
2		2	2													
1		1	1													
2		2	2													
1		1	1													
2		2	2													
1		1	1													
2		2	2													
1		1	1													
2		2	2													
1		1	1													
2		2	2													
Total Xpert Positive:				Total Rif Resistance		Total tested Positive:	Total MDR-TB:	Total number suggestive:	Total number testedpositive:	Total number reactive:	Total:	Total started Rx:	Total died:	Total not on Rx:	Total started TPT:	

6. Tick whether sputum results were back within 48hrs.

7. Write date on which the patient was initiated on Treatment.

8. Write a date for TPT start.

Complied by: .... Signature: .... Date: ....

Verified by: .... Signature: .... Date: ....

health
Department:
Health
REPUBLIC OF SOUTH AFRICA

TUBERCULOSIS IDENTIFICATION REGISTER

District: ....

									Risk Groups				TB Investigations: Bacteriological investigation: BASELINE TESTS	
Specimen Barcode Number[1]	Patient Folder Number	Surname (Top row)<br>First name(s) (Bottom row)	Date of birth (Top row) DD / MM / YYYY<br>Age (Bottom row)	Gender M / F	Physical Home Address[2] / Land Mark	Telephone / Cellphone	TB symptoms Identified<br>Yes / No<br>If Yes (Code)[3]	Contact (Y / N) (Top row)<br>DS/ DR- TB / unk (Bottom row)	HIV Status (Pos / Neg / Unk)	Pregnant (Y / N)	Diabetes Mellitus (Y / N / Unk)	COVID-19 (Pos / Neg / Unk)	Date Specimen collected[4] DD / MM / YYYY	Date test Result received[5] DD / MM / YYYY
													1	1
													2	2
													1	1
													2	2
													1	1
													2	2
													1	1
													2	2
													1	1
													2	2
													1	1
													2	2
													1	1
													2	2
													1	1
													2	2
													1	1
													2	2
													1	1
													2	2
TOTALS:									Total number positive:	Total number pregnant:	Total number Diabetic:	Total number positive:		

1. Place Barcode sticker from the laboratory form / write Barcode number eg. AAGX8459

2. Full detailed address of where the patient lives or can be reached during the day.

3. Use the coding on the instruction cover for TB symptoms (i.e. 1= Cough)

4. Write down a date the patient produced specimen

5: Write date when the test results were received by facility

Sub District: .... Facility Name: .... 2020 Version GW20/13

TB Investigations													TB Confirmed Clients (DS-TB and DR-TB)			TPT Start Date[8] (for contacts, PLHIV) DD / MM / YYYY	REMARKS
Bacteriological investigation									Non- Bacteriological Investigations								
**Xpert** (1st Test)	**Smear Microscopy** (Pos, Neg, Scanty, not done / no result)	**TEST RESULT TURN AROUND TIME[6]** (Tick)		**Rif Susceptibility**	**TB CULTURE / LPA / DST**			**X-Ray** DD / MM / YYYY	**U-LAM** DD / MM / YYYY	**Mantoux** DD / MM / YYYY	**Other Tests** DD / MM / YYYY	**Date Treatment Started[7]** DD / MM / YYYY	**Died before Treatment Start** (Tick)	**Loss to follow up** (Tick)			
**Xpert** (2nd test if any)	**Grading** (bottom row)	< 48hrs	> 48hrs	R / S	**Date specimen collected** DD / MM / YYYY	**Result** (Pos / Neg)	**Resistance** (R / H)	Result	Result	Result	Result						
1		1	1														
2		2	2														
1		1	1														
2		2	2														
1		1	1														
2		2	2														
1		1	1														
2		2	2														
1		1	1														
2		2	2														
1		1	1														
2		2	2														
1		1	1														
2		2	2														
1		1	1														
2		2	2														
1		1	1														
2		2	2														
1		1	1														
2		2	2														
Total Xpert Positive:				Total Rif Resistance		Total tested Positive:	Total MDR-TB:	Total number suggestive:	Total number testedpositive:	Total number reactive:	Total:	Total started Rx:	Total died:	Total not on Rx:	Total started TPT:		

6. Tick whether sputum results were back within 48hrs.

7. Write date on which the patient was initiated on Treatment.

8. Write a date for TPT start.

Complied by: .... Signature: .... Date: .... Verified by: .... Signature: .... Date: ....

health
Department:
Health
REPUBLIC OF SOUTH AFRICA

TUBERCULOSIS IDENTIFICATION REGISTER

District: ....

								Risk Groups					TB Investigations	
													Bacteriological investigation	
													BASELINE TESTS	
**Specimen Barcode Number**[1]	**Patient Folder Number**	**Surname** (Top row) **First name(s)** (Bottom row)	**Date of birth** (Top row) DD / MM / YYYY **Age** (Bottom row)	**Gender M / F**	**Physical Home Address**[2] **/ Land Mark**	**Telephone / Cellphone**	**TB symptoms Identified** **Yes / No** **If Yes (Code)**[3]	**Contact (Y / N)** (Top row) **DS/ DR- TB / unk** (Bottom row)	**HIV Status** (Pos / Neg / Unk)	**Pregnant** (Y / N)	**Diabetes Mellitus** (Y / N / Unk)	**COVID-19** (Pos / Neg / Unk)	**Date Specimen collected**[4] DD / MM / YYYY	**Date test Result received**[5] DD / MM / YYYY
													1	1
													2	2
													1	1
													2	2
													1	1
													2	2
													1	1
													2	2
													1	1
													2	2
													1	1
													2	2
													1	1
													2	2
													1	1
													2	2
													1	1
													2	2
													1	1
													2	2
TOTALS:									Total number positive:	Total number pregnant:	Total number Diabetic:	Total number positive:		

1. Place Barcode sticker from the laboratory form / write Barcode number eg. AAGX8459

2. Full detailed address of where the patient lives or can be reached during the day.

3. Use the coding on the instruction cover for TB symptoms (i.e. 1= Cough)

4. Write down a date the patient produced specimen

5: Write date when the test results were received by facility

Sub District: .... Facility Name: .... 2020 Version GW20/13

TB Investigations												TB Confirmed Clients (DS-TB and DR-TB)			TPT Start Date[8] (for contacts, PLHIV) DD / MM / YYYY	REMARKS
Bacteriological investigation								Non- Bacteriological Investigations								
**Xpert** (1st Test)	**Smear Microscopy** (Pos, Neg, Scanty, not done / no result)	**TEST RESULT TURN AROUND TIME[6]** (Tick)		**Rif Susceptibility**	**TB CULTURE / LPA / DST**			**X-Ray** DD / MM / YYYY	**U-LAM** DD / MM / YYYY	**Mantoux** DD / MM / YYYY	**Other Tests** DD / MM / YYYY	**Date Treatment Started[7]** DD / MM / YYYY	**Died before Treatment Start** (Tick)	**Loss to follow up** (Tick)		
**Xpert** (2nd test if any)	**Grading** (bottom row)	< 48hrs	> 48hrs	R / S	**Date specimen collected** DD / MM / YYYY	**Result** (Pos / Neg)	**Resistance** (R / H)	Result	Result	Result	Result					
1		1	1													
2		2	2													
1		1	1													
2		2	2													
1		1	1													
2		2	2													
1		1	1													
2		2	2													
1		1	1													
2		2	2													
1		1	1													
2		2	2													
1		1	1													
2		2	2													
1		1	1													
2		2	2													
1		1	1													
2		2	2													
1		1	1													
2		2	2													
Total Xpert Positive:				Total Rif Resistance		Total tested Positive:	Total MDR-TB:	Total number suggestive:	Total number testedpositive:	Total number reactive:	Total:	Total started Rx:	Total died:	Total not on Rx:	Total started TPT:	

6. Tick whether sputum results were back within 48hrs.

7. Write date on which the patient was initiated on Treatment.

8. Write a date for TPT start.

Complied by: .... Signature: .... Date: .... Verified by: .... Signature: .... Date: ....

health
Department:
Health
REPUBLIC OF SOUTH AFRICA

TUBERCULOSIS IDENTIFICATION REGISTER

District: ....

Specimen Barcode Number[1]	Patient Folder Number	Surname (Top row)<br>First name(s) (Bottom row)	Date of birth (Top row) DD / MM / YYYY<br>Age (Bottom row)	Gender M / F	Physical Home Address[2] / Land Mark	Telephone / Cellphone	TB symptoms Identified	Risk Groups					TB Investigations	
							Yes / No<br>If Yes (Code)[3]	Contact (Y / N) (Top row)<br>DS/ DR- TB / unk (Bottom row)	HIV Status (Pos / Neg / Unk)	Pregnant (Y / N)	Diabetes Mellitus (Y / N / Unk)	COVID-19 (Pos / Neg / Unk)	Bacteriological investigation<br>BASELINE TESTS<br>Date Specimen collected[4] DD / MM / YYYY	Date test Result received[5] DD / MM / YYYY
													1	1
													2	2
													1	1
													2	2
													1	1
													2	2
													1	1
													2	2
													1	1
													2	2
													1	1
													2	2
													1	1
													2	2
													1	1
													2	2
													1	1
													2	2
													1	1
													2	2
TOTALS:									Total number positive:	Total number pregnant:	Total number Diabetic:	Total number positive:		

1. Place Barcode sticker from the laboratory form / write Barcode number eg. AAGX8459

2. Full detailed address of where the patient lives or can be reached during the day.

3. Use the coding on the instruction cover for TB symptoms (i.e. 1= Cough)

4. Write down a date the patient produced specimen

5: Write date when the test results were received by facility

Sub District: .... Facility Name: ....

TB Investigations												TB Confirmed Clients (DS-TB and DR-TB)			TPT Start Date[8] (for contacts, PLHIV) DD / MM / YYYY	REMARKS
Bacteriological investigation								Non- Bacteriological Investigations								
**Xpert** (1st Test)	**Smear Microscopy** (Pos, Neg, Scanty, not done / no result)	**TEST RESULT TURN AROUND TIME[6]** (Tick)		**Rif Susceptibility**	**TB CULTURE / LPA / DST**			**X-Ray** DD / MM / YYYY	**U-LAM** DD / MM / YYYY	**Mantoux** DD / MM / YYYY	**Other Tests** DD / MM / YYYY	**Date Treatment Started[7]** DD / MM / YYYY	**Died before Treatment Start** (Tick)	**Loss to follow up** (Tick)		
**Xpert** (2nd test if any)	**Grading** (bottom row)	< 48hrs	> 48hrs	R / S	**Date specimen collected** DD / MM / YYYY	**Result** (Pos / Neg)	**Resistance** (R / H)	Result	Result	Result	Result					
1		1	1													
2		2	2													
1		1	1													
2		2	2													
1		1	1													
2		2	2													
1		1	1													
2		2	2													
1		1	1													
2		2	2													
1		1	1													
2		2	2													
1		1	1													
2		2	2													
1		1	1													
2		2	2													
1		1	1													
2		2	2													
Total Xpert Positive:				Total Rif Resistance		Total tested Positive:	Total MDR-TB:	Total number suggestive:	Total number testedpositive:	Total number reactive:	Total:	Total started Rx:	Total died:	Total not on Rx:	Total started TPT:	

6. Tick whether sputum results were back within 48hrs.

7. Write date on which the patient was initiated on Treatment.

8. Write a date for TPT start.

Complied by: .... Signature: .... Date: ....

Verified by: .... Signature: .... Date: ....

health
Department:
Health
REPUBLIC OF SOUTH AFRICA

TUBERCULOSIS IDENTIFICATION REGISTER

District: ....

Specimen Barcode Number[1]	Patient Folder Number	Surname (Top row) / First name(s) (Bottom row)	Date of birth (Top row) DD / MM / YYYY / Age (Bottom row)	Gender M / F	Physical Home Address[2] / Land Mark	Telephone / Cellphone	TB symptoms Identified: Yes / No (Top row) / If Yes (Code)[3] (Bottom row)	Risk Groups: Contact (Y / N) (Top row) / DS/ DR- TB / unk (Bottom row)	Risk Groups: HIV Status (Pos / Neg / Unk)	Risk Groups: Pregnant (Y / N)	Risk Groups: Diabetes Mellitus (Y / N / Unk)	Risk Groups: COVID-19 (Pos / Neg / Unk)	TB Investigations – Bacteriological investigation – BASELINE TESTS: Date Specimen collected[4] DD / MM / YYYY	TB Investigations – Bacteriological investigation – BASELINE TESTS: Date test Result received[5] DD / MM / YYYY
													1	1
													2	2
													1	1
													2	2
													1	1
													2	2
													1	1
													2	2
													1	1
													2	2
													1	1
													2	2
													1	1
													2	2
													1	1
													2	2
													1	1
													2	2
													1	1
													2	2
TOTALS:									Total number positive:	Total number pregnant:	Total number Diabetic:	Total number positive:		

1. Place Barcode sticker from the laboratory form / write Barcode number eg. AAGX8459

2. Full detailed address of where the patient lives or can be reached during the day.

3. Use the coding on the instruction cover for TB symptoms (i.e. 1= Cough)

4. Write down a date the patient produced specimen

5: Write date when the test results were received by facility

Sub District: .... Facility Name: ....

TB Investigations												TB Confirmed Clients (DS-TB and DR-TB)			TPT Start Date[8] (for contacts, PLHIV) DD / MM / YYYY	REMARKS
Bacteriological investigation								Non- Bacteriological Investigations								
**Xpert** (1st Test)	**Smear Microscopy** (Pos, Neg, Scanty, not done / no result)	**TEST RESULT TURN AROUND TIME[6]** (Tick)		**Rif Susceptibility**	**TB CULTURE / LPA / DST**			**X-Ray** DD / MM / YYYY	**U-LAM** DD / MM / YYYY	**Mantoux** DD / MM / YYYY	**Other Tests** DD / MM / YYYY	**Date Treatment Started[7]** DD / MM / YYYY	**Died before Treatment Start** (Tick)	**Loss to follow up** (Tick)		**REMARKS**
**Xpert** (2nd test if any)	**Grading** (bottom row)	< 48hrs	> 48hrs	R / S	**Date specimen collected** DD / MM / YYYY	**Result** (Pos / Neg)	**Resistance** (R / H)	Result	Result	Result	Result					
1		1	1													
2		2	2													
1		1	1													
2		2	2													
1		1	1													
2		2	2													
1		1	1													
2		2	2													
1		1	1													
2		2	2													
1		1	1													
2		2	2													
1		1	1													
2		2	2													
1		1	1													
2		2	2													
1		1	1													
2		2	2													
1		1	1													
2		2	2													
Total Xpert Positive:				Total Rif Resistance		Total tested Positive:	Total MDR-TB:	Total number suggestive:	Total number testedpositive:	Total number reactive:	Total:	Total started Rx:	Total died:	Total not on Rx:	Total started TPT:	

6. Tick whether sputum results were back within 48hrs.

7. Write date on which the patient was initiated on Treatment.

8. Write a date for TPT start.

Complied by: .... Signature: .... Date: .... Verified by: .... Signature: .... Date: ....

health
Department:
Health
REPUBLIC OF SOUTH AFRICA

TUBERCULOSIS IDENTIFICATION REGISTER

District: ....

Specimen Barcode Number[1]	Patient Folder Number	Surname (Top row) / First name(s) (Bottom row)	Date of birth (Top row) DD / MM / YYYY / Age (Bottom row)	Gender M / F	Physical Home Address[2] / Land Mark	Telephone / Cellphone	TB symptoms Identified: Yes / No (Top row) / If Yes (Code)[3] (Bottom row)	Risk Groups: Contact (Y / N) (Top row) / DS/ DR- TB / unk (Bottom row)	Risk Groups: HIV Status (Pos / Neg / Unk)	Risk Groups: Pregnant (Y / N)	Risk Groups: Diabetes Mellitus (Y / N / Unk)	Risk Groups: COVID-19 (Pos / Neg / Unk)	TB Investigations – Bacteriological investigation – BASELINE TESTS: Date Specimen collected[4] DD / MM / YYYY	TB Investigations – Bacteriological investigation – BASELINE TESTS: Date test Result received[5] DD / MM / YYYY
													1	1
													2	2
													1	1
													2	2
													1	1
													2	2
													1	1
													2	2
													1	1
													2	2
													1	1
													2	2
													1	1
													2	2
													1	1
													2	2
													1	1
													2	2
													1	1
													2	2
TOTALS:									Total number positive:	Total number pregnant:	Total number Diabetic:	Total number positive:		

1. Place Barcode sticker from the laboratory form / write Barcode number eg. AAGX8459

2. Full detailed address of where the patient lives or can be reached during the day.

3. Use the coding on the instruction cover for TB symptoms (i.e. 1= Cough)

4. Write down a date the patient produced specimen

5: Write date when the test results were received by facility

Sub District: .... Facility Name: .... 2020 Version GW20/13

TB Investigations												TB Confirmed Clients (DS-TB and DR-TB)			TPT Start Date[8] (for contacts, PLHIV) DD / MM / YYYY	REMARKS
Bacteriological investigation								Non- Bacteriological Investigations								
Xpert (1st Test)	Smear Microscopy (Pos, Neg, Scanty, not done / no result)	TEST RESULT TURN AROUND TIME[6] (Tick)		Rif Susceptibility	TB CULTURE / LPA / DST			X-Ray DD / MM / YYYY	U-LAM DD / MM / YYYY	Mantoux DD / MM / YYYY	Other Tests DD / MM / YYYY	Date Treatment Started[7] DD / MM / YYYY	Died before Treatment Start (Tick)	Loss to follow up (Tick)		
Xpert (2nd test if any)	Grading (bottom row)	< 48hrs	> 48hrs	R / S	Date specimen collected DD / MM / YYYY	Result (Pos / Neg)	Resistance (R / H)	Result	Result	Result	Result					
1		1	1													
2		2	2													
1		1	1													
2		2	2													
1		1	1													
2		2	2													
1		1	1													
2		2	2													
1		1	1													
2		2	2													
1		1	1													
2		2	2													
1		1	1													
2		2	2													
1		1	1													
2		2	2													
1		1	1													
2		2	2													
1		1	1													
2		2	2													
Total Xpert Positive:				Total Rif Resistance		Total tested Positive:	Total MDR-TB:	Total number suggestive:	Total number testedpositive:	Total number reactive:	Total:	Total started Rx:	Total died:	Total not on Rx:	Total started TPT:	

6. Tick whether sputum results were back within 48hrs.

7. Write date on which the patient was initiated on Treatment.

8. Write a date for TPT start.

Complied by: .... Signature: .... Date: ....

Verified by: .... Signature: .... Date: ....

health
Department:
Health
REPUBLIC OF SOUTH AFRICA

TUBERCULOSIS IDENTIFICATION REGISTER

District: ....

Specimen Barcode Number[1]	Patient Folder Number	Surname (Top row) / First name(s) (Bottom row)	Date of birth (Top row) DD / MM / YYYY / Age (Bottom row)	Gender M / F	Physical Home Address[2] / Land Mark	Telephone / Cellphone	TB symptoms Identified: Yes / No / If Yes (Code)[3]	Risk Groups: Contact (Y / N) (Top row) / DS/ DR- TB / unk (Bottom row)	Risk Groups: HIV Status (Pos / Neg / Unk)	Risk Groups: Pregnant (Y / N)	Risk Groups: Diabetes Mellitus (Y / N / Unk)	Risk Groups: COVID-19 (Pos / Neg / Unk)	TB Investigations – Bacteriological investigation – BASELINE TESTS: Date Specimen collected[4] DD / MM / YYYY	TB Investigations – Bacteriological investigation – BASELINE TESTS: Date test Result received[5] DD / MM / YYYY
													1	1
													2	2
													1	1
													2	2
													1	1
													2	2
													1	1
													2	2
													1	1
													2	2
													1	1
													2	2
													1	1
													2	2
													1	1
													2	2
													1	1
													2	2
													1	1
													2	2
TOTALS:									Total number positive:	Total number pregnant:	Total number Diabetic:	Total number positive:		

1. Place Barcode sticker from the laboratory form / write Barcode number eg. AAGX8459

2. Full detailed address of where the patient lives or can be reached during the day.

3. Use the coding on the instruction cover for TB symptoms (i.e. 1= Cough)

4. Write down a date the patient produced specimen

5: Write date when the test results were received by facility

Sub District: .... Facility Name: ....

2020 Version
GW20/13

TB Investigations												TB Confirmed Clients (DS-TB and DR-TB)			TPT Start Date[8] (for contacts, PLHIV) DD / MM / YYYY	REMARKS
Bacteriological investigation								Non- Bacteriological Investigations								
**Xpert** (1st Test)	**Smear Microscopy** (Pos, Neg, Scanty, not done / no result)	**TEST RESULT TURN AROUND TIME[6]** (Tick)		**Rif Susceptibility**	**TB CULTURE / LPA / DST**			**X-Ray** DD / MM / YYYY	**U-LAM** DD / MM / YYYY	**Mantoux** DD / MM / YYYY	**Other Tests** DD / MM / YYYY	**Date Treatment Started[7]** DD / MM / YYYY	**Died before Treatment Start** (Tick)	**Loss to follow up** (Tick)		
**Xpert** (2nd test if any)	**Grading** (bottom row)	< 48hrs	> 48hrs	R / S	**Date specimen collected** DD / MM / YYYY	**Result** (Pos / Neg)	**Resistance** (R / H)	Result	Result	Result	Result					
1		1	1													
2		2	2													
1		1	1													
2		2	2													
1		1	1													
2		2	2													
1		1	1													
2		2	2													
1		1	1													
2		2	2													
1		1	1													
2		2	2													
1		1	1													
2		2	2													
1		1	1													
2		2	2													
1		1	1													
2		2	2													
1		1	1													
2		2	2													
Total Xpert Positive:				Total Rif Resistance		Total tested Positive:	Total MDR-TB:	Total number suggestive:	Total number testedpositive:	Total number reactive:	Total:	Total started Rx:	Total died:	Total not on Rx:	Total started TPT:	

6. Tick whether sputum results were back within 48hrs.

7. Write date on which the patient was initiated on Treatment.

8. Write a date for TPT start.

Complied by: .... Signature: .... Date: .... Verified by: .... Signature: .... Date: ....

health
Department: Health
REPUBLIC OF SOUTH AFRICA

TUBERCULOSIS IDENTIFICATION REGISTER

District: ....

								Risk Groups					TB Investigations	
													Bacteriological investigation	
													BASELINE TESTS	
Specimen Barcode Number[1]	Patient Folder Number	Surname (Top row) First name(s) (Bottom row)	Date of birth (Top row) DD / MM / YYYY Age (Bottom row)	Gender M / F	Physical Home Address[2] / Land Mark	Telephone / Cellphone	TB symptoms Identified Yes / No If Yes (Code)[3]	Contact (Y / N) (Top row) DS/ DR- TB / unk (Bottom row)	HIV Status (Pos / Neg / Unk)	Pregnant (Y / N)	Diabetes Mellitus (Y / N / Unk)	COVID-19 (Pos / Neg / Unk)	Date Specimen collected[4] DD / MM / YYYY	Date test Result received[5] DD / MM / YYYY
													1	1
													2	2
													1	1
													2	2
													1	1
													2	2
													1	1
													2	2
													1	1
													2	2
													1	1
													2	2
													1	1
													2	2
													1	1
													2	2
													1	1
													2	2
													1	1
													2	2
TOTALS:									Total number positive:	Total number pregnant:	Total number Diabetic:	Total number positive:		

1. Place Barcode sticker from the laboratory form / write Barcode number eg. AAGX8459

2. Full detailed address of where the patient lives or can be reached during the day.

3. Use the coding on the instruction cover for TB symptoms (i.e. 1= Cough)

4. Write down a date the patient produced specimen

5: Write date when the test results were received by facility

Sub District: .... Facility Name: ....

2020 Version
GW20/13

TB Investigations												TB Confirmed Clients (DS-TB and DR-TB)			TPT Start Date[8] (for contacts, PLHIV) DD / MM / YYYY	REMARKS
Bacteriological investigation								Non- Bacteriological Investigations								
**Xpert** (1st Test)	**Smear Microscopy** (Pos, Neg, Scanty, not done / no result)	**TEST RESULT TURN AROUND TIME[6]** (Tick)		**Rif Susceptibility**	**TB CULTURE / LPA / DST**			**X-Ray** DD / MM / YYYY	**U-LAM** DD / MM / YYYY	**Mantoux** DD / MM / YYYY	**Other Tests** DD / MM / YYYY	**Date Treatment Started[7]** DD / MM / YYYY	**Died before Treatment Start** (Tick)	**Loss to follow up** (Tick)		
**Xpert** (2nd test if any)	**Grading** (bottom row)	< 48hrs	> 48hrs	R / S	**Date specimen collected** DD / MM / YYYY	**Result** (Pos / Neg)	**Resistance** (R / H)	Result	Result	Result	Result					
1		1	1													
2		2	2													
1		1	1													
2		2	2													
1		1	1													
2		2	2													
1		1	1													
2		2	2													
1		1	1													
2		2	2													
1		1	1													
2		2	2													
1		1	1													
2		2	2													
1		1	1													
2		2	2													
1		1	1													
2		2	2													
1		1	1													
2		2	2													
Total Xpert Positive:				Total Rif Resistance		Total tested Positive:	Total MDR-TB:	Total number suggestive:	Total number testedpositive:	Total number reactive:	Total:	Total started Rx:	Total died:	Total not on Rx:	Total started TPT:	

6. Tick whether sputum results were back within 48hrs.

7. Write date on which the patient was initiated on Treatment.

8. Write a date for TPT start.

Complied by: .... Signature: .... Date: .... Verified by: .... Signature: .... Date: ....

health
Department: Health
REPUBLIC OF SOUTH AFRICA

TUBERCULOSIS IDENTIFICATION REGISTER

District: ....

Specimen Barcode Number[1]	Patient Folder Number	Surname (Top row) / First name(s) (Bottom row)	Date of birth (Top row) DD / MM / YYYY / Age (Bottom row)	Gender M / F	Physical Home Address[2] / Land Mark	Telephone / Cellphone	TB symptoms Identified: Yes / No / If Yes (Code)[3]	Risk Groups: Contact (Y / N) (Top row) / DS/ DR- TB / unk (Bottom row)	Risk Groups: HIV Status (Pos / Neg / Unk)	Risk Groups: Pregnant (Y / N)	Risk Groups: Diabetes Mellitus (Y / N / Unk)	Risk Groups: COVID-19 (Pos / Neg / Unk)	TB Investigations – Bacteriological investigation – BASELINE TESTS: Date Specimen collected[4] DD / MM / YYYY	TB Investigations – Bacteriological investigation – BASELINE TESTS: Date test Result received[5] DD / MM / YYYY
													1	1
													2	2
													1	1
													2	2
													1	1
													2	2
													1	1
													2	2
													1	1
													2	2
													1	1
													2	2
													1	1
													2	2
													1	1
													2	2
													1	1
													2	2
													1	1
													2	2
TOTALS:									Total number positive:	Total number pregnant:	Total number Diabetic:	Total number positive:		

1. Place Barcode sticker from the laboratory form / write Barcode number eg. AAGX8459

2. Full detailed address of where the patient lives or can be reached during the day.

3. Use the coding on the instruction cover for TB symptoms (i.e. 1= Cough)

4. Write down a date the patient produced specimen

5: Write date when the test results were received by facility

Sub District: .... Facility Name: ....

TB Investigations												TB Confirmed Clients (DS-TB and DR-TB)				
Bacteriological investigation								Non- Bacteriological Investigations								
**Xpert** (1st Test)	**Smear Microscopy** (Pos, Neg, Scanty, not done / no result)	**TEST RESULT TURN AROUND TIME**[6] (Tick)		**Rif Susceptibility**	**TB CULTURE / LPA / DST**			**X-Ray** DD / MM / YYYY	**U-LAM** DD / MM / YYYY	**Mantoux** DD / MM / YYYY	**Other Tests** DD / MM / YYYY	**Date Treatment Started**[7] DD / MM / YYYY	**Died before Treatment Start** (Tick)	**Loss to follow up** (Tick)	**TPT Start Date**[8] (for contacts, PLHIV) DD / MM / YYYY	**REMARKS**
**Xpert** (2nd test if any)	**Grading** (bottom row)	< 48hrs	> 48hrs	R / S	**Date specimen collected** DD / MM / YYYY	**Result** (Pos / Neg)	**Resistance** (R / H)	Result	Result	Result	Result					
1		1	1													
2		2	2													
1		1	1													
2		2	2													
1		1	1													
2		2	2													
1		1	1													
2		2	2													
1		1	1													
2		2	2													
1		1	1													
2		2	2													
1		1	1													
2		2	2													
1		1	1													
2		2	2													
1		1	1													
2		2	2													
1		1	1													
2		2	2													
Total Xpert Positive:				Total Rif Resistance		Total tested Positive:	Total MDR-TB:	Total number suggestive:	Total number testedpositive:	Total number reactive:	Total:	Total started Rx:	Total died:	Total not on Rx:	Total started TPT:	

6. Tick whether sputum results were back within 48hrs.

7. Write date on which the patient was initiated on Treatment.

8. Write a date for TPT start.

Complied by: .... Signature: .... Date: .... Verified by: .... Signature: .... Date: ....

health
Department:
Health
REPUBLIC OF SOUTH AFRICA

TUBERCULOSIS IDENTIFICATION REGISTER

District: ....

Specimen Barcode Number[1]	Patient Folder Number	Surname (Top row) / First name(s) (Bottom row)	Date of birth (Top row) DD / MM / YYYY / Age (Bottom row)	Gender M / F	Physical Home Address[2] / Land Mark	Telephone / Cellphone	TB symptoms Identified: Yes / No / If Yes (Code)[3]	Risk Groups: Contact (Y / N) (Top row) / DS/ DR- TB / unk (Bottom row)	Risk Groups: HIV Status (Pos / Neg / Unk)	Risk Groups: Pregnant (Y / N)	Risk Groups: Diabetes Mellitus (Y / N / Unk)	Risk Groups: COVID-19 (Pos / Neg / Unk)	TB Investigations – Bacteriological investigation – BASELINE TESTS: Date Specimen collected[4] DD / MM / YYYY	TB Investigations – Bacteriological investigation – BASELINE TESTS: Date test Result received[5] DD / MM / YYYY
													1	1
													2	2
													1	1
													2	2
													1	1
													2	2
													1	1
													2	2
													1	1
													2	2
													1	1
													2	2
													1	1
													2	2
													1	1
													2	2
													1	1
													2	2
													1	1
													2	2
TOTALS:									Total number positive:	Total number pregnant:	Total number Diabetic:	Total number positive:		

1. Place Barcode sticker from the laboratory form / write Barcode number eg. AAGX8459

2. Full detailed address of where the patient lives or can be reached during the day.

3. Use the coding on the instruction cover for TB symptoms (i.e. 1= Cough)

4. Write down a date the patient produced specimen

5: Write date when the test results were received by facility

Sub District: .... Facility Name: ....

2020 Version
GW20/13

TB Investigations												TB Confirmed Clients (DS-TB and DR-TB)			**TPT Start Date[8]** (for contacts, PLHIV) DD / MM / YYYY	REMARKS
Bacteriological investigation								Non- Bacteriological Investigations								
**Xpert** (1st Test)	**Smear Microscopy** (Pos, Neg, Scanty, not done / no result)	**TEST RESULT TURN AROUND TIME[6]** (Tick)		**Rif Susceptibility**	**TB CULTURE / LPA / DST**			**X-Ray** DD / MM / YYYY	**U-LAM** DD / MM / YYYY	**Mantoux** DD / MM / YYYY	**Other Tests** DD / MM / YYYY	**Date Treatment Started[7]** DD / MM / YYYY	**Died before Treatment Start** (Tick)	**Loss to follow up** (Tick)		
**Xpert** (2nd test if any)	**Grading** (bottom row)	< 48hrs	> 48hrs	R / S	**Date specimen collected** DD / MM / YYYY	**Result** (Pos / Neg)	**Resistance** (R / H)	Result	Result	Result	Result					
1		1	1													
2		2	2													
1		1	1													
2		2	2													
1		1	1													
2		2	2													
1		1	1													
2		2	2													
1		1	1													
2		2	2													
1		1	1													
2		2	2													
1		1	1													
2		2	2													
1		1	1													
2		2	2													
1		1	1													
2		2	2													
1		1	1													
2		2	2													
Total Xpert Positive:				Total Rif Resistance		Total tested Positive:	Total MDR-TB:	Total number suggestive:	Total number testedpositive:	Total number reactive:	Total:	Total started Rx:	Total died:	Total not on Rx:	Total started TPT:	

6. Tick whether sputum results were back within 48hrs.

7. Write date on which the patient was initiated on Treatment.

8. Write a date for TPT start.

Complied by: .... Signature: .... Date: ....

Verified by: .... Signature: .... Date: ....

health
Department:
Health
REPUBLIC OF SOUTH AFRICA

TUBERCULOSIS IDENTIFICATION REGISTER

District: ....

Specimen Barcode Number[1]	Patient Folder Number	Surname (Top row) First name(s) (Bottom row)	Date of birth (Top row) DD / MM / YYYY	Gender M / F	Physical Home Address[2] / Land Mark	Telephone / Cellphone	TB symptoms Identified	Risk Groups					TB Investigations	
							Yes / No	Contact (Y / N) (Top row)	HIV Status (Pos / Neg / Unk)	Pregnant (Y / N)	Diabetes Mellitus (Y / N / Unk)	COVID-19 (Pos / Neg / Unk)	Bacteriological investigation	
			Age (Bottom row)				If Yes (Code)[3]	DS/ DR- TB / unk (Bottom row)					BASELINE TESTS	
													Date Specimen collected[4] DD / MM / YYYY	Date test Result received[5] DD / MM / YYYY
													1	1
													2	2
													1	1
													2	2
													1	1
													2	2
													1	1
													2	2
													1	1
													2	2
													1	1
													2	2
													1	1
													2	2
													1	1
													2	2
													1	1
													2	2
													1	1
													2	2
TOTALS:									Total number positive:	Total number pregnant:	Total number Diabetic:	Total number positive:		

1. Place Barcode sticker from the laboratory form / write Barcode number eg. AAGX8459

2. Full detailed address of where the patient lives or can be reached during the day.

3. Use the coding on the instruction cover for TB symptoms (i.e. 1= Cough)

4. Write down a date the patient produced specimen

5: Write date when the test results were received by facility

Sub District: .... Facility Name: ....

2020 Version
GW20/13

TB Investigations												TB Confirmed Clients (DS-TB and DR-TB)			TPT Start Date[8] (for contacts, PLHIV) DD / MM / YYYY	REMARKS
Bacteriological investigation								Non- Bacteriological Investigations								
**Xpert** (1st Test)	**Smear Microscopy** (Pos, Neg, Scanty, not done / no result)	**TEST RESULT TURN AROUND TIME[6]** (Tick)		**Rif Susceptibility**	**TB CULTURE / LPA / DST**			**X-Ray** DD / MM / YYYY	**U-LAM** DD / MM / YYYY	**Mantoux** DD / MM / YYYY	**Other Tests** DD / MM / YYYY	**Date Treatment Started[7]** DD / MM / YYYY	**Died before Treatment Start** (Tick)	**Loss to follow up** (Tick)		**REMARKS**
**Xpert** (2nd test if any)	**Grading** (bottom row)	< 48hrs	> 48hrs	R / S	**Date specimen collected** DD / MM / YYYY	**Result** (Pos / Neg)	**Resistance** (R / H)	Result	Result	Result	Result					
1		1	1													
2		2	2													
1		1	1													
2		2	2													
1		1	1													
2		2	2													
1		1	1													
2		2	2													
1		1	1													
2		2	2													
1		1	1													
2		2	2													
1		1	1													
2		2	2													
1		1	1													
2		2	2													
1		1	1													
2		2	2													
1		1	1													
2		2	2													
Total Xpert Positive:				Total Rif Resistance		Total tested Positive:	Total MDR-TB:	Total number suggestive:	Total number testedpositive:	Total number reactive:	Total:	Total started Rx:	Total died:	Total not on Rx:	Total started TPT:	

6. Tick whether sputum results were back within 48hrs.

7. Write date on which the patient was initiated on Treatment.

8. Write a date for TPT start.

Complied by: .... Signature: .... Date: ....

Verified by: .... Signature: .... Date: ....

health
Department:
Health
REPUBLIC OF SOUTH AFRICA

TUBERCULOSIS IDENTIFICATION REGISTER

District: ....

Specimen Barcode Number[1]	Patient Folder Number	Surname (Top row)<br>First name(s) (Bottom row)	Date of birth (Top row) DD / MM / YYYY<br>Age (Bottom row)	Gender M / F	Physical Home Address[2] / Land Mark	Telephone / Cellphone	TB symptoms Identified<br>Yes / No<br>If Yes (Code)[3]	Risk Groups: Contact (Y / N) (Top row)<br>DS/ DR- TB / unk (Bottom row)	Risk Groups: HIV Status (Pos / Neg / Unk)	Risk Groups: Pregnant (Y / N)	Risk Groups: Diabetes Mellitus (Y / N / Unk)	Risk Groups: COVID-19 (Pos / Neg / Unk)	TB Investigations – Bacteriological investigation – BASELINE TESTS: Date Specimen collected[4] DD / MM / YYYY	TB Investigations – Bacteriological investigation – BASELINE TESTS: Date test Result received[5] DD / MM / YYYY
													1	1
													2	2
													1	1
													2	2
													1	1
													2	2
													1	1
													2	2
													1	1
													2	2
													1	1
													2	2
													1	1
													2	2
													1	1
													2	2
													1	1
													2	2
													1	1
													2	2
TOTALS:									Total number positive:	Total number pregnant:	Total number Diabetic:	Total number positive:		

1. Place Barcode sticker from the laboratory form / write Barcode number eg. AAGX8459

2. Full detailed address of where the patient lives or can be reached during the day.

3. Use the coding on the instruction cover for TB symptoms (i.e. 1= Cough)

4. Write down a date the patient produced specimen

5: Write date when the test results were received by facility

Sub District: .... Facility Name: .... 2020 Version GW20/13

TB Investigations												TB Confirmed Clients (DS-TB and DR-TB)			TPT Start Date[8] (for contacts, PLHIV) DD / MM / YYYY	REMARKS
Bacteriological investigation								Non- Bacteriological Investigations								
**Xpert** (1st Test)	**Smear Microscopy** (Pos, Neg, Scanty, not done / no result)	**TEST RESULT TURN AROUND TIME[6]** (Tick)		**Rif Susceptibility**	**TB CULTURE / LPA / DST**			**X-Ray** DD / MM / YYYY	**U-LAM** DD / MM / YYYY	**Mantoux** DD / MM / YYYY	**Other Tests** DD / MM / YYYY	**Date Treatment Started[7]** DD / MM / YYYY	**Died before Treatment Start** (Tick)	**Loss to follow up** (Tick)		
**Xpert** (2nd test if any)	**Grading** (bottom row)	< 48hrs	> 48hrs	R / S	**Date specimen collected** DD / MM / YYYY	**Result** (Pos / Neg)	**Resistance** (R / H)	Result	Result	Result	Result					
1		1	1													
2		2	2													
1		1	1													
2		2	2													
1		1	1													
2		2	2													
1		1	1													
2		2	2													
1		1	1													
2		2	2													
1		1	1													
2		2	2													
1		1	1													
2		2	2													
1		1	1													
2		2	2													
1		1	1													
2		2	2													
Total Xpert Positive:				Total Rif Resistance		Total tested Positive:	Total MDR-TB:	Total number suggestive:	Total number testedpositive:	Total number reactive:	Total:	Total started Rx:	Total died:	Total not on Rx:	Total started TPT:	

6. Tick whether sputum results were back within 48hrs.

7. Write date on which the patient was initiated on Treatment.

8. Write a date for TPT start.

Complied by: .... Signature: .... Date: .... Verified by: .... Signature: .... Date: ....

health
Department:
Health
REPUBLIC OF SOUTH AFRICA

TUBERCULOSIS IDENTIFICATION REGISTER

District: ....

Specimen Barcode Number[1]	Patient Folder Number	Surname (Top row) First name(s) (Bottom row)	Date of birth (Top row) DD / MM / YYYY	Gender M / F	Physical Home Address[2] / Land Mark	Telephone / Cellphone	TB symptoms Identified	Risk Groups					TB Investigations	
							Yes / No	Contact (Y / N) (Top row)	HIV Status (Pos / Neg / Unk)	Pregnant (Y / N)	Diabetes Mellitus (Y / N / Unk)	COVID-19 (Pos / Neg / Unk)	Bacteriological investigation	
			Age (Bottom row)				If Yes (Code)[3]	DS/ DR- TB / unk (Bottom row)					BASELINE TESTS	
													Date Specimen collected[4] DD / MM / YYYY	Date test Result received[5] DD / MM / YYYY
													1	1
													2	2
													1	1
													2	2
													1	1
													2	2
													1	1
													2	2
													1	1
													2	2
													1	1
													2	2
													1	1
													2	2
													1	1
													2	2
													1	1
													2	2
													1	1
													2	2
TOTALS:									Total number positive:	Total number pregnant:	Total number Diabetic:	Total number positive:		

1. Place Barcode sticker from the laboratory form / write Barcode number eg. AAGX8459

2. Full detailed address of where the patient lives or can be reached during the day.

3. Use the coding on the instruction cover for TB symptoms (i.e. 1= Cough)

4. Write down a date the patient produced specimen

5: Write date when the test results were received by facility

Sub District: .... Facility Name: .... 2020 Version GW20/13

TB Investigations												TB Confirmed Clients (DS-TB and DR-TB)			TPT Start Date[8] (for contacts, PLHIV) DD / MM / YYYY	REMARKS
Bacteriological investigation								Non- Bacteriological Investigations								
Xpert (1st Test)	Smear Microscopy (Pos, Neg, Scanty, not done / no result)	TEST RESULT TURN AROUND TIME[6] (Tick)		Rif Susceptibility	TB CULTURE / LPA / DST			X-Ray DD / MM / YYYY	U-LAM DD / MM / YYYY	Mantoux DD / MM / YYYY	Other Tests DD / MM / YYYY	Date Treatment Started[7] DD / MM / YYYY	Died before Treatment Start (Tick)	Loss to follow up (Tick)		
Xpert (2nd test if any)	Grading (bottom row)	< 48hrs	> 48hrs	R / S	Date specimen collected DD / MM / YYYY	Result (Pos / Neg)	Resistance (R / H)	Result	Result	Result	Result					
1		1	1													
2		2	2													
1		1	1													
2		2	2													
1		1	1													
2		2	2													
1		1	1													
2		2	2													
1		1	1													
2		2	2													
1		1	1													
2		2	2													
1		1	1													
2		2	2													
1		1	1													
2		2	2													
1		1	1													
2		2	2													
1		1	1													
2		2	2													
Total Xpert Positive:				Total Rif Resistance		Total tested Positive:	Total MDR-TB:	Total number suggestive:	Total number testedpositive:	Total number reactive:	Total:	Total started Rx:	Total died:	Total not on Rx:	Total started TPT:	

6. Tick whether sputum results were back within 48hrs.

7. Write date on which the patient was initiated on Treatment.

8. Write a date for TPT start.

Complied by: .... Signature: .... Date: .... Verified by: .... Signature: .... Date: ....

health
Department:
Health
REPUBLIC OF SOUTH AFRICA

TUBERCULOSIS IDENTIFICATION REGISTER

District: ....

Specimen Barcode Number[1]	Patient Folder Number	Surname (Top row) / First name(s) (Bottom row)	Date of birth (Top row) DD / MM / YYYY / Age (Bottom row)	Gender M / F	Physical Home Address[2] / Land Mark	Telephone / Cellphone	TB symptoms Identified	Risk Groups					TB Investigations	
							Yes / No (Top row) / If Yes (Code)[3] (Bottom row)	Contact (Y / N) (Top row) / DS/ DR- TB / unk (Bottom row)	HIV Status (Pos / Neg / Unk)	Pregnant (Y / N)	Diabetes Mellitus (Y / N / Unk)	COVID-19 (Pos / Neg / Unk)	Bacteriological investigation — BASELINE TESTS: Date Specimen collected[4] DD / MM / YYYY	Bacteriological investigation — BASELINE TESTS: Date test Result received[5] DD / MM / YYYY
													1	1
													2	2
													1	1
													2	2
													1	1
													2	2
													1	1
													2	2
													1	1
													2	2
													1	1
													2	2
													1	1
													2	2
													1	1
													2	2
													1	1
													2	2
													1	1
													2	2
TOTALS:									Total number positive:	Total number pregnant:	Total number Diabetic:	Total number positive:		

1. Place Barcode sticker from the laboratory form / write Barcode number eg. AAGX8459

2. Full detailed address of where the patient lives or can be reached during the day.

3. Use the coding on the instruction cover for TB symptoms (i.e. 1= Cough)

4. Write down a date the patient produced specimen

5: Write date when the test results were received by facility

Sub District: .... Facility Name: ....

2020 Version
GW20/13

TB Investigations													TB Confirmed Clients (DS-TB and DR-TB)			TPT Start Date[8] (for contacts, PLHIV) DD / MM / YYYY	REMARKS
Bacteriological investigation								Non- Bacteriological Investigations									
Xpert (1st Test)	Smear Microscopy (Pos, Neg, Scanty, not done / no result)	TEST RESULT TURN AROUND TIME[6] (Tick)		Rif Susceptibility	TB CULTURE / LPA / DST			X-Ray DD / MM / YYYY	U-LAM DD / MM / YYYY	Mantoux DD / MM / YYYY	Other Tests DD / MM / YYYY	Date Treatment Started[7] DD / MM / YYYY	Died before Treatment Start (Tick)	Loss to follow up (Tick)		
Xpert (2nd test if any)	Grading (bottom row)	< 48hrs	> 48hrs	R / S	Date specimen collected DD / MM / YYYY	Result (Pos / Neg)	Resistance (R / H)	Result	Result	Result	Result					
1		1	1													
2		2	2													
1		1	1													
2		2	2													
1		1	1													
2		2	2													
1		1	1													
2		2	2													
1		1	1													
2		2	2													
1		1	1													
2		2	2													
1		1	1													
2		2	2													
1		1	1													
2		2	2													
1		1	1													
2		2	2													
1		1	1													
2		2	2													
Total Xpert Positive:				Total Rif Resistance		Total tested Positive:	Total MDR-TB:	Total number suggestive:	Total number testedpositive:	Total number reactive:	Total:	Total started Rx:	Total died:	Total not on Rx:	Total started TPT:	

6. Tick whether sputum results were back within 48hrs.

7. Write date on which the patient was initiated on Treatment.

8. Write a date for TPT start.

Complied by: .... Signature: .... Date: ....

Verified by: .... Signature: .... Date: ....

health

Department:
Health
REPUBLIC OF SOUTH AFRICA

SUMMARY FOR TB DETECTION

Year:		Number of clients with TB symptoms			Risk groups with identified TB symptoms					Risk groups Investigated for TB					Number of clients investigated for TB			
		Total	> 5 yrs	≤ 5 yrs	HIV Pos	Diabetic	Pregnant	Contacts	COVID 19	HIV Pos	Diabetic	Pregnant	Contacts	COVID 19	Bacteriological tests		Non Bacteriological tests	
															< 5 yrs	≥ 5 yrs	< 5 yrs	≥ 5 yrs
Quarter 1	January																	
	February																	
	March																	
	**Total**																	
Quarter 2	April																	
	May																	
	June																	
	**Total**																	
Quarter 3	July																	
	August																	
	September																	
	**Total**																	
Quarter 4	October																	
	November																	
	December																	
	**Total**																	

TB Confirmed Risk Amongst Risk Groups					Results of TB Investigations				Outcomes for TB confirmed clients					Number of Contacts with negative investigation initiated on TPT		Number of specimen sent to lab for testing (Xpert)	Number of test results (Xpert) received within 48 hrs (2 days)
HIV Pos	Diabetic	Pregnant	Contacts	COVID 19	Bacteriologically confirmed		Clinically Confirmed		Started on TB Treatment		Other Outcomes						
					< 5 yrs	≥ 5 yrs	< 5 yrs	≥ 5 yrs	< 5 yrs	≥ 5 yrs	RR	Died	LTFU	< 5 yrs	≥ 5 yrs		

health

Department:
Health
REPUBLIC OF SOUTH AFRICA

NTCP TB Identification Register 1020 V9.indd 44 2020/10/06 1:51 PM
