# Supplementary material for: Assessment of facility-based tuberculosis data quality in an integrated HIV/TB database in three South African districts
Source: PLOS Glob Public Health. 2022 Sep 28;2(9):e0000312. doi: 10.1371/journal.pgph.0000312 (PMC10021242; doi:10.1371/journal.pgph.0000312)
Supplement: S2 File — (PDF) [file pgph.0000312.s002.pdf]

HOUSEHOLD CONTACTS

Name of contact	Age	Symptom Screened		Treatment started			
				IPT		TB	
		Yes	No	Yes	No	Yes	No
		Yes	No	Yes	No	Yes	No
		Yes	No	Yes	No	Yes	No
		Yes	No	Yes	No	Yes	No
		Yes	No	Yes	No	Yes	No
		Yes	No	Yes	No	Yes	No
		Yes	No	Yes	No	Yes	No
		Yes	No	Yes	No	Yes	No
		Yes	No	Yes	No	Yes	No
		Yes	No	Yes	No	Yes	No

TREATMENT OUTCOMES

Treatment stop date: d d m m y y y y

- [ ] **Cured**
- [ ] **Treatment Completed**
- [ ] **Lost to follow up**
- [ ] **Failed treatment** [ ] **MDR-TB** [ ] **Rif Resistant TB**
- [ ] **Died**

REFERRALS

[ ] Moved out [ ] Transferred out Date: d d m m y y y y

Name of receiving clinic: ______

Town/ District: ______

Province/ Country: ______

Discharged by (Print name): ______

G.PS. 004-2575

GW 20/12
2018

health
Department:
Health
REPUBLIC OF SOUTH AFRICA

TB TREATMENT RECORD

TB Registration Number:

Name:

Surname:

Facility Name: ______ District: ______

Patient Folder Number: ______

N	Newly Registered in this facility
M	Moved in from facility in this district
T	Transferred in from another district

Facility Name: ______

TB Reg No: ______

PATIENT DETAILS

ID Number/Date of birth: y y m m d d _ _ _ _ _ _ _ Age ____ Gender M F

PHYSICAL ADDRESS

Home Address:	Name of Company/ Employer:
	Work address:
Tel No./Cellphone:	Tel No:

PATIENT CATEGORY

- [ ] New
- [ ] Relapse
- [ ] Re-treatment after Loss to follow up
- [ ] Re-treatment after Failure
- [ ] Other Previously Treated

- [ ] Rifampicin susceptible TB

CLASSIFICATION OF DISEASE

ICD10 Code ______

Pulmonary TB [ ]

Extra Pulmonary TB [ ]

Site of disease ______

[ ] Isoniazid resistant TB

TREATMENT REGIMEN

[ ] Regimen 1 [ ] Regimen 3 [ ] Other Specify: ______

Treatment Start Date d d m m y y y y

NEXT OF KIN or FRIEND DETAILS

Surname ______ First Name(s) ______ Phone Number ______

Address: ______

NOTIFICATION INFORMATION (GW17/5)

Has the GW17/5 form been completed? Y N Notification date: d d m m y y y y

XPERT, LINE PROBE ASSAY, CULTURE, DST RESULTS

GENEXPERT						
Date	Result			Rifampicin Susceptibility Results		
	Positive	Negative	Unsuccessful	Resistant	Susceptible	Unsuccessful
	Positive	Negative	Unsuccessful	Resistant	Susceptible	Unsuccessful
	Positive	Negative	Unsuccessful	Resistant	Susceptible	Unsuccessful

LINE PROBE ASSAY (LPA)							
Date	Result			Drug Susceptibility Results			
				Rifampicin		Isoniazid	
	Positive	Negative	Contaminated	Resistant	Susceptible	Resistant	Susceptible
	Positive	Negative	Contaminated	Resistant	Susceptible	Resistant	Susceptible

CULTURE							
Date	Culture Result			Drug Susceptibility Test (DST) Results			
				Rifampicin		Isoniazid	
	Positive	Negative	Contaminated	Resistant	Susceptible	Resistant	Susceptible
	Positive	Negative	Contaminated	Resistant	Susceptible	Resistant	Susceptible

SMEAR MICROSCOPY RESULTS

Date	Baseline		End of continuation phase				End of treatment	
			7 weeks		11 weeks		23 weeks	
	Result		Result		Result		Result	
	Positive	Negative	Positive	Negative	Negative	Positive	Negative	Positive
	Positive	Negative	Positive	Negative	Negative	Positive	Negative	Positive
	Positive	Negative	Positive	Negative	Negative	Positive	Negative	Positive
	Positive	Negative	Positive	Negative	Negative	Positive	Negative	Positive

OTHER DIAGNOSTIC TESTS CONDUCTED

X-rays: ______________________________

Other tests conducted/ performed: ______________________________

Findings: ______________________________

Test results: ______________________________

MEDICAL HISTORY

KNOWN MEDICAL CONDITIONS			CURRENT MEDICATION
**Hypertension**	Yes	No	
**Diabetes**	Yes	No	
**Epilepsy**	Yes	No	
**Mental illness**	Yes	No	
**Liver disease**	Yes	No	
**Renal insufficiency**	Yes	No	
**Allergies (specify)**			
**Other (specify)**			

Last menstrual period: ______________________ **Contraceptive method:** ____________________

HIV INFORMATION

**HIV Status**	Pos	Neg	Unk
**On Cotrimoxazole**	Yes	No	N/A
**On ART**	Yes	No	N/A

HIV Test conducted: **Yes** **No** **If yes, result:** __________
(Circle where applicable)

If on ART, Regimen: ______________________________

RISK FACTORS

Tobacco use	Pos	Neg
Alcohol use	Yes	No
Substance use	Yes	No

Assess for severity

Has the patient ever:

Worked in a mine	Yes	No
Spent time in prison	Yes	No
Been admitted in hospital	Yes	No

(Refer to occupational health clinic/ MBOD if ex mineworker)

TREATMENT

INTENSIVE PHASE (IP)

Body weight at start of IP [ ] kg

Medicine	RHZE (150/75/400/275)	*RHZ (75/50/150)	RH (60/60)	R	H	Z	E	S
**No. of tablets/ dosage per day**								

* New formulation

Month	Date 1	2	3	4	5	6	7	8	9	10	11	12	13	14	15	16	17	18	19	20	21	22	23	24	25	26	27	28	29	30	31	No.of Doses taken

Ö = patient took medication
x = patient did not take medication
— = medication collected for self administration or supervision elsewhere

CONTINUATION PHASE (CP)

Body weight at start of CP [ ] kg

Medicine	RH (150/75)	RH (300/150)	*RH (75/50)	RH (60/60)	R	H	E
**Number of tablets/ dosage per day**							

* New formulation

Month	Date 1	2	3	4	5	6	7	8	9	10	11	12	13	14	15	16	17	18	19	20	21	22	23	24	25	26	27	28	29	30	31	No.of Doses taken

Ö = patient took medication
x = patient did not take medication
— = medication collected for self administration or supervision elsewhere
