# Supplementary material for: Assessment of facility-based tuberculosis data quality in an integrated HIV/TB database in three South African districts
Source: PLOS Glob Public Health. 2022 Sep 28;2(9):e0000312. doi: 10.1371/journal.pgph.0000312 (PMC10021242; doi:10.1371/journal.pgph.0000312)
Supplement: S1 Table — (DOCX) [file pgph.0000312.s003.docx]

**S1 Table:** Description of data elements and how we captured them from TIER.Net

| **Data element** | **How it was coded** | **Comment** |
| --- | --- | --- |
| Name-match  Surname-match  Date of birth-match  Gender-match  Folder number-match | Yes/No based-on data capturers verification | We did not collect identifiers in REDCap so they were not included in our analytical dataset. |
| Referral Form | Yes/No | There was no clear comparable field in TIER.Net. |
| Service Point:  TB Service Point  ART Service | Multiple-choice | “Service Point” was not a field on the “GW 20/12 2018” TB Treatment Record (blue file) and generally not available in most of the clinical files we reviewed. |
| Registration type – TIER.Net  Newly registered  Transferred/Moved-in | Multiple-choice | We did not record the 3^rd^ category available on the TB Treatment Record – “Transferred in from another district”. |
| Patient Category  New  Relapse  Retreatment after 1st line failure  Retreatment after default | Multiple-choice | TIER.Net only allows a single entry) while the TB Treatment Record allows multiple categories to be selected. |
| TB treatment Regimen*  Regimen 1  Regimen 3  Other | Multiple-choice | - |
| TB Treatment Start Date | Date | - |
| Disease class  Extra pulmonary TB  Pulmonary TB | Multiple-choice | - |
| Site of disease | Multiple choice | Site of disease in the TB Treatment Record was an open text field while TIER.Net has multiple choice options for site of disease which are aligned to disease class. For example, “A18.5 Tuberculosis of the eye” would only be available to select for Site of disease in TIER.Net if “Extra pulmonary TB” was selected from Disease class while you there’s no “controls” on the TB Treatment Record a paper record. |
| Laboratory tests  Smear Microscopy (TB sputum)  GeneXpert MTB/RIF Assay  X - Ray  Culture  LPA  Other | We recorded each type of test as well as dates for up to:   - 4 smears - 3 GeneXpert results - 2 cultures - 2 LPAs | The first set of questions was a checklist  In some cases, no labs were recorded on both or either record. |
| Visit dates | - Total visits - Visit dates | We recorded up to 12 specific visit dates per TIER.Net record/TB Treatment Record. |
| TB Treatment Outcome  Lost to follow-up  Died  Transferred/Moved out  RIF resistance case  MDR case  Cured  Treatment completed  Treatment failure  Missing/unknown/not assigned | Multiple-choice | - |
| HIV Status  Positive  Negative  Unknown | Multiple-choice |  |
