# Supplementary material for: Assessment of facility-based tuberculosis data quality in an integrated HIV/TB database in three South African districts
Source: PLOS Glob Public Health. 2022 Sep 28;2(9):e0000312. doi: 10.1371/journal.pgph.0000312 (PMC10021242; doi:10.1371/journal.pgph.0000312)
Supplement: S2 Table — (DOCX) [file pgph.0000312.s004.docx]

**S2 Table:** Site of disease completeness

| **Variable** | **TB Treatment Record:**  **n (%)** | **TIER.Net: n (%)** | **P value*** |
| --- | --- | --- | --- |
| **N** | 214 | 222 |  |
| **Site of disease** | | |  |
| TB of lung, confirmed by sputum microscopy with or without culture (A15.0) | 14/154 (9%) | 80/211 (38%) | **<0.001** |
| TB of lung, confirmed by culture only (A15.1) | 71/154 (46%) | 6/211 (3%) |  |
| TB of lung, confirmed by unspecified means (A15.3) | 10/154 (6%) | 21/211 (10%) |  |
| Recorded as “Other” | 15/154 (10%) | 5/211 (2%) |  |
| TB of lung, bacteriological and histological examination not done (A16.1) | 0/154 (0%) | 20/211 (9%) |  |
| Acute miliary TB of a single specified site (A19.0) | 8/154 (5%) | 8/211 (4% |  |
| TB of lung, without mention of bacteriological or histological confirmation (A16.2) | 0/154 (0%) | 16/211 (8%) |  |
| TB of lung, bacteriologically and histologically negative (A16.0) | 0/154 (0%) | 15/211 (7%) |  |
| Other respiratory TB, without mention of bacteriological or histological confirmation (A16.8) | 6/154 (4%) | 7/211 (3%) |  |
| Respiratory TB unspecified, without mention of bacteriological or histological confirmation (A16.9) | 7/154 (5%) | 5/211 (2%) |  |
| TB of lung, confirmed histologically (A15.2) | 5/154 (3%) | 3/211 (1%) |  |
| TB of bones and joints (A18.0) | 4/154 (3%) | 3/211 (1%) |  |
| TB peripheral lymphadenopathy (A18.2) | 3/154 (2%) | 4/211 (2%) |  |
| TB of intra thoracic lymph nodes, confirmed bacteriologically and histologically (A15.4) | 2/154 (1%) | 3/211 (1%) |  |
| TB of other specified organs (A18.8) | 3/154 (2%) | 2/211 (1%) |  |
| Other codes | 8 different site of disease codes often with only a single example | 13 different site of disease codes often with only a single example |  |
