# Supplementary material for: Assessment of facility-based tuberculosis data quality in an integrated HIV/TB database in three South African districts
Source: PLOS Glob Public Health. 2022 Sep 28;2(9):e0000312. doi: 10.1371/journal.pgph.0000312 (PMC10021242; doi:10.1371/journal.pgph.0000312)
Supplement: S3 Table — (DOCX) [file pgph.0000312.s005.docx]

**S3 Table:** 2x2 tables indicating proportion of agreement: first GeneXpert result (n=98) and first smear result (n=112).

|  | | **Clinic record first GeneXpert result** | | | 75 positive results in TIER.Net matched the 76 positive results in the clinic record (99%).  While 17 negative results in TIER.Net matched the 22 in the clinic record (77% of cases) |
| --- | --- | --- | --- | --- | --- |
|  |  | Positive | Negative | **Total** |  |
| **TIER.Net first GeneXpert results – earliest** | Positive | **75** | 5 | 80 |  |
|  | Negative | 1 | **17** | 18 |  |
|  | **Total** | 76 | 22 | 98 |  |
|  | | **Clinic record first smear result** | | | 24 positive results in TIER.Net matched the 31 positive results in the clinic record (77%).  While 74 negative results in TIER.Net matched the 81 in the clinic record (91% of cases) |
|  |  | Positive | Negative | **Total** |  |
| **TIER.Net first smear**  **result** | Positive | **24** | 7 | 31 |  |
|  | Negative | 7 | **74** | 81 |  |
|  | **Total** | 31 | 81 | 112 |  |

**Supplement B: 2x2 tables indicating proportion of agreement: final TB outcome successful/unsuccessful (n=163).**

|  | | **TB treatment outcome in TB Treatment Record** | | | 140 successful outcomes TIER.Net matched the 144 successful outcomes in the clinic record (97%).  While 16 unsuccessful outcomes matched 19 in the clinic record (84% of cases) |
| --- | --- | --- | --- | --- | --- |
|  |  | Successful | Unsuccessful | **Total** |  |
| **TB treatment outcome in TIER.Net** | Successful | **140** | 3 | 143 |  |
|  | Unsuccessful | 4 | **16** | 20 |  |
|  | **Total** | 144 | 19 | 163 |  |
