# Supplementary material for: Assessment of facility-based tuberculosis data quality in an integrated HIV/TB database in three South African districts
Source: PLOS Glob Public Health. 2022 Sep 28;2(9):e0000312. doi: 10.1371/journal.pgph.0000312 (PMC10021242; doi:10.1371/journal.pgph.0000312)
Supplement: Attachment — Submitted filename: Review Responses_PGPH-D-22-00374-PLOSGH 11Aug2022.DOCX [file pgph.0000312.s006.DOCX]

11 August 2022

To: PLOS Global Health Editors

Re: Revision of our manuscript **PGPH-D-22-00374**

Dear Editorial Team,

I am pleased to resubmit the attached manuscript, **“Assessment of facility-based tuberculosis data quality in an integrated HIV/TB database in three South African districts.”** for consideration in PLOS Global Health.

We have addressed the editor’s advice and each reviewer’s comments and revised the manuscript. We are grateful for the constructive feedback and attention to detail given by each of the reviewers, and believe that the manuscript is much stronger and clearer now. The point-by-point response to the editor’s and reviewers’ comments can be found below and we have also attached a tracked changes version of the manuscript and tables, so that the editors and reviewers can easily see the changes we have made. We have also attached a clean version of the revised manuscript.

To summarize, we have included 3 attachments:

1. “Response to reviewers”: A rebuttal letter that responds to each point raised by the editor and reviewer(s) (in closed bullet). Our responses are below each request with an open bullet in blue font. (this very document)
2. “Revised manuscript with track changes”: A marked-up copy of the revised manuscript that highlights changes made to the original version.
3. “Manuscript”: An unmarked version of the revised paper without tracked changes.

We thank the editors and reviewers for their consideration of this revised manuscript.

Yours sincerely,

|  |  |
| --- | --- |

Joshua Murphy

Researcher, HE^2^RO

NIH Fogarty Global Health Fellow

E-mail: [joshpmurphy@gmail.com](mailto:joshpmurphy@gmail.com)

**Response to Editor’s comments:**

- Please provide separate figure files in .tif or .eps format and remove the embedded figures within the manuscript file.
- Authors’ response: Thank you, yes, we have done this
- Since your data is not available for proprietary reasons, please explain via email why the data is not available. Please also include the contact information for the third-party organization that should be contacted should other researchers want to request access to this data and please include the full citation of where the data can be found.
- Authors’ response: Thank you, we will send a copy of this full response in an email separately, below is the full response with key information documenting why data are not available.
- The datasets generated and/or analyzed during the current study is owned by the South African Government and use is governed by the University of the Witwatersrand Human Research Ethics Committee (HREC – Medical). Data cannot be shared publicly because the local ethics committee restricts access. Access to these data is subject to restrictions owing to privacy and ethics policies set by the South African Government, so they are not publicly available.
- Requests can be directed to: Health Economics and Epidemiology Research Office (HE2RO) Ross Greener [rgreener@heroza.org](mailto:rgreener@heroza.org) HE2RO’s Head of Data or [information@heroza.org](mailto:information@heroza.org)
- Human Research Ethics Committee (HREC) at the University of Witwatersrand Professor Clement Penny, +27 11 717 2301, or by e-mail on [Clement.Penny@wits.ac.za](mailto:Clement.Penny@wits.ac.za). The telephone numbers for the Committee secretariat are +27 11 717 2700/1234 and the e-mail addresses are [Zanele.Ndlovu@wits.ac.za](mailto:Zanele.Ndlovu@wits.ac.za) and [Rhulani.Mukansi@wits.ac.za](mailto:Rhulani.Mukansi@wits.ac.za)
- We also request that you verify with us via email that any researcher will be able to obtain the data set in the same manner that the you have obtained it. If you feel you are unwilling or unable to adhere to this policy, please explain your reasons by return email and your exemption request will be escalated to the editor for approval.
- Authors’ response: We can confirm that any researcher, who follows the same process that we followed, will be able to replicate the dataset that was generated and analyzed for this study
  - Your exemption request will be handled independently and will not hold up the peer review process, but will need to be resolved should your manuscript be accepted for publication. One of the Editorial team will be in touch if they require more information.
- Authors’ response: Thank you; this is clear.
  - Please review your reference list to ensure that it is complete and correct. If you have cited papers that have been retracted, please include the rationale for doing so in the manuscript text, or remove these references and replace them with relevant current references. Any changes to the reference list should be mentioned in the rebuttal letter that accompanies your revised manuscript. If you need to cite a retracted article, indicate the article’s retracted status in the References list and also include a citation and full reference for the retraction notice.
- Authors’ response: Thank you; yes, we have conducted a final review of the reference list. A few detailed changes are documented below.
- Note line numbers below are based on the tracked changed version.

**Response to Reviewer comments:**

**1.** Does this manuscript meet PLOS Global Public Health’s publication criteria? Is the manuscript technically sound, and do the data support the conclusions? The manuscript must describe methodologically and ethically rigorous research with conclusions that are appropriately drawn based on the data presented.

Reviewer #1: Yes

Reviewer #2: Yes

**2.** Has the statistical analysis been performed appropriately and rigorously?

Reviewer #1: Yes

Reviewer #2: Yes

- Authors’ response: Thank you., again we are happy to hear this.

________________________________________

**3.** Have the authors made all data underlying the findings in their manuscript fully available (please refer to the Data Availability Statement at the start of the manuscript PDF file)? The PLOS Data policy requires authors to make all data underlying the findings described in their manuscript fully available without restriction, with rare exception. The data should be provided as part of the manuscript or its supporting information, or deposited to a public repository. For example, in addition to summary statistics, the data points behind means, medians and variance measures should be available. If there are restrictions on publicly sharing data—e.g. participant privacy or use of data from a third party—those must be specified.

Reviewer #1: No

Reviewer #2: No

4. Is the manuscript presented in an intelligible fashion and written in standard English? PLOS Global Public Health does not copyedit accepted manuscripts, so the language in submitted articles must be clear, correct, and unambiguous. Any typographical or grammatical errors should be corrected at revision, so please note any specific errors here.

Reviewer #1: No

Reviewer #2: No

**Reviewer #1:** This manuscript describes a retrospective evaluation of data quality of the TB module in TIER.Net in three South African districts. I have few comments for the authors to consider and/or clarify.

- Lines 67-69: “We report the quality of TB data captured in an electronic HIV/TB database, the Three Interlinked Electronic Register (TIER.Net), to contribute to this understanding.” This read as study aim/objective. I suggest moving this sentence to end of the introduction or rephrasing it.
  - Authors’ response: Thank you very much. This is a helpful solution. As such, we decided to remove that sentence (**Line 68**) as it amounted to a repeat of the final sentence of the introduction. We had to make a small change on **Line 73** to update the description of the TIER.Net acronym.
  - **Lines 109 -111:** The authors conducted data quality assessment of the TB module in TIER.Net alongside a set of informal qualitative interviews to understand the strengths and weaknesses of the TB module in three districts of South Africa. What is meant by “set of informal qualitative interviews”? How these “set of informal qualitative interviews” were analysed? Why are the results not presented in the results section and discussed in the discussion section?
  - Authors’ response: Thank you for highlighting this oversight. As this manuscript focuses on the quantitative assessment of data quality, we decided to remove mention of this (**Line 110**).
  - **Lines 112 -117:** The authors selected three districts in consultation with the South African NDoH Drug-sensitive (DS)-TB Directorate and National TB-Think Tank, however it is unclear why these three districts were selected.
  - Authors’ response: Thank you for the opportunity to clarify this. Ultimately, we were limited by time and resources. We had existing relationships at these districts as well as staff that were able to access these areas. Fortunately, Gauteng was representative of higher TB-burden metropolitan areas while the other two district/provinces were representative of rural and peri-urban areas across South Africa.
  - The following sentence has been added to **Line 116**: “Districts and facilities were selected based on the following criteria: (1) TB cases diagnosed and treatment start rate, identified from the routine District Health Information System (DHIS) and District Health Barometer 2018/2019 (17). We selected one high volume province (Gauteng) and two low volume provinces (North West and Limpopo). Within these, we selected one district above the treatment start rate (Bojanala Platinum 108.0 versus SA average 97.8) and one below the treatment start rate (Mopani 95.2 versus SA average 97.8). Johannesburg was below the average at 89.2., (2) the environment, including urban (Johannesburg), per-urban (Bojanala Platinum) or rural (Mopani), (3) whether we had existing relationships with facilities or districts, and last (4) whether we had staff that was familiar with these areas and could move through these areas easily.”
  - **Line 157:** The authors used Stata and MS Excel for analysis. Why was MS Excel used for analysis?
- Authors’ response: MS Excel was primarily used as a study record management tool and not for analysis. We would like to exclude MS Excel with this in mind. We therefore cut this from **Line 171** “Stata version 15 was used for analysis.”
  - **Line 463:** References need to formatted to follow a specific referencing style, especially WHO and governmental documents
  - Authors’ response: Thank you; yes, we have conducted a final review of the reference list.
  - **Line 513 (noting lines numbers are changing),** The author was rather South African National Department of Health and now WHO, as they served as technical leaders of the work. The reference now reads:

1. South Africa National Department of Health. Joint Review of HIV, TB and PMTCT Programmes in South Africa | Department of Health Knowledge Hub [Internet]. 2014 [cited 2022 Aug 10]. Available from <https://www.knowledgehub.org.za/elibrary/joint-review-hiv-tb-and-pmtct-programmes-south-africa>

- **Line 531** is now corrected as: 5. World Health Organization. End TB by 2030. WHO Libr Cat Data [Internet]. 2016 [cited 2022 Aug 10];1–28. Available from: <https://apps.who.int/iris/bitstream/handle/10665/259636/TBstrat-eng.pdf?sequence=1>
- We also updated URLs for references on **line 552, 556, 564, 567**

**Reviewer #2:** The study compared TB records in South Africa across 3 sources - the TB treatment record or patient medical file; the TB Identification Register; and the TB module in TIER.Net – the electronic source.

- The Goal was to evaluate data quality/ concordance of Tier.net data with the facility-based patient records and the ETR.net
- Sampling:- logistical feasibility was described as the major reason for selecting the sites.
- Data collection between October 2021 and December 2021;
- Study population /patient records from October 18 -December 2019.
- Objectives: Describe the sample ; the accuracy of demographic information – agreement between sources; completeness of Tb-related information; accuracy of key TB-related vars and outcomes – agreement between measurements; lab results agreement
- Analytical methods: Kappa statistics for agreement, Mann–Whitney/Wilcoxon rank-sum test to compare independent samples and non-parametric continuous data; Chi-squared tests for independence of categorical variable.
- Results: The distribution of demographic characteristics of patients across districts was fairly similar. The authors found Tier.net to be more complete for some key variables and they attribute that to some of the built-in features (e.g. logic checks) within the electronic db as compared to pare-based records.
- General comment: This is a valuable contribution to TB-related research. Data quality [completeness and accuracy] is crucial for guiding TB programs. The manuscript is well-written overall, with some areas that need to be improved.

**Comments**

- **1. The abstract mentions three** approaches that were used to make comparisons but these are not explicitly described in the methods section and it leaves the reader wondering. Could the abstract simply say something like “the comparisons were done with each of the three sources as a starting point. Throughout I found myself wondering what these “approaches” are.
- Authors’ response: Thank you for highlighting this. This sentence has been corrected and **Line 31 - 35** now reads:
- “using three sources and three approaches to link these (i.e., two approaches compared TIER.NET with the TB Treatment Record while the third approach compared all three sources of TB data: the TB treatment record or patient medical file; the TB Identification Register; and the TB module in TIER.Net). We report agreement and completeness of demographic information and key TB-related variables across all three data sources.”
- **2. Sampling –** more details have to be provided about the selection of the 15 facilities. How were they situated across the 3 districts? Was a random approach used to select them?
- Authors’ response: Thank you very much for this question. Convenience sampling was used as South Africa has a generalized TB epidemic and the available literature suggests TB data quality is consistently sub-optimal. Five facilities were selected in each district based.

**On Line 116** we have added the following to address this key omission: “Districts and facilities were selected based on the following criteria: (1) TB cases diagnosed and treatment start rate, identified from the routine District Health Information System (DHIS) and District Health Barometer 2018/2019 (17). We selected one high volume province (Gauteng) and two low volume provinces (North West and Limpopo). Within these, we selected one district above the treatment start rate (Bojanala Platinum 108.0 versus SA average 97.8) and one below the treatment start rate (Mopani 95.2 versus SA average 97.8). Johannesburg was below the average at 89.2., (2) the environment, including urban (Johannesburg), per-urban (Bojanala Platinum) or rural (Mopani), (3) whether we had existing relationships with facilities or districts, and last (4) whether we had staff that was familiar with these areas and could move through these areas easily.”

- **3. Table 2 needs some clarification** – it is not clear what the “Sum of DS-TB cases...” is. Is this the number of patient files available for this time period from which the study sample was selected? What is the relationship between the first row and the rest of the rows?
- Authors’ response: Agreed, thanks again for this. The intention of the first row is to explain the differences across districts in terms of patient load and TB burden. The time periods overlap the TB treatment start dates in our sample, however, it is primarily for illustrative purposes. We will remove the first row of the table (**Line 247**) to address this concern and we believe the critical aspect has been addressed through **Line 116** mentioned above.
- **4**. **For key TB-related variables in Table 4**, the authors conducted global tests of independence which indicates whether the patterns for reporting match between sources in general. This however does not give an exact evaluation of whether the values of these variables do match between sources. This is not as helpful as the results in Table 5 which lists agreement (the one-to-one correspondence check) between sources.
- Authors’ response: Thanks for this helpful observation. The intention is to compare **completeness of the information** between the two types of records at a global level. So agreed, Table 5 provides a more helpful **measure of accuracy**. However, there is still value to show the completeness of each source. Is there any specific action that will better address this important comment?
  - **5. Within facility** – a form of systematic sampling was used to select 10 records with treatment date closest to study dates of interest. It is mentioned that files that met eligibility criteria were selected but it is not clear what the eligibility criteria were.
  - Authors’ response: Thanks for this very helpful point. As a solution we have added the following on **Line 133:** “Eligibility criteria included: aged 18 years or older, DS-TB only and (not extensively drug-resistant TB nor multidrug-resistant tuberculosis), and a TB treatment start date in the timeframe above.” And we therefore removed the 18 years and older point on **Line 229.**
  - We also clarified sampling procedures on **Line 135:**
    - “Records were collected consecutively, according to the abovementioned criteria and the procedures outlined under “Data Sources”, and working backward until the required number of records had been collected.”
  - 6. PLOS authors have the option to publish the peer review history of their article (what does this mean?). If published, this will include your full peer review and any attached files.
  - Do you want your identity to be public for this peer review? If you choose “no”, your identity will remain anonymous but your review may still be made public.
  - For information about this choice, including consent withdrawal, please see our Privacy Policy.

Reviewer #1: No

Reviewer #2: Yes: Bareng A.S.Nonyane

________________________________________

- While revising your submission, please upload your figure files to the Preflight Analysis and Conversion Engine (PACE) digital diagnostic tool, https://pacev2.apexcovantage.com/. PACE helps ensure that figures meet PLOS requirements. To use PACE, you must first register as a user. Registration is free. Then, login and navigate to the UPLOAD tab, where you will find detailed instructions on how to use the tool. If you encounter any issues or have any questions when using PACE, please email PLOS at figures@plos.org. Please note that Supporting Information files do not need this step.
- Authors’ response: Perfect, we will follow this process. Thanks again!

Notes on upload process:
